# 3-Methyl-4*H*-chromen-4-one

**DOI:** 10.1107/S1600536810020453

**Published:** 2010-06-05

**Authors:** Lujiang Hao, Jiangkui Chen, Xiaofei Zhang

**Affiliations:** a Shandong Provincial Key Laboratory of Microbial Engineering, Shandong Institute of Light Industry, Jinan 250353, People’s Republic of China

## Abstract

In the title chromenone derivative, C_10_H_8_O_2_, the two fused six-membered rings are coplanar, with a mean deviation of 0.0261 (1) Å from the plane through the non-H atoms of the rings. The carbonyl and methyl substituents of the pyran ring also lie close to that plane, with the O and C atoms deviating by 0.0557 (1) and 0.1405 (1) Å, respectively. In the crystal, weak C—H⋯O contacts form chains along the *a* axis.

## Related literature

For the pharmaceutical applications of chromanone compounds, see: Shi *et al.* (2004 ▶). For related structures, see: Takikawa & Suzuki (2007 ▶); Patonay *et al.* (2002 ▶); Alaniz & Rovis, (2005 ▶). 
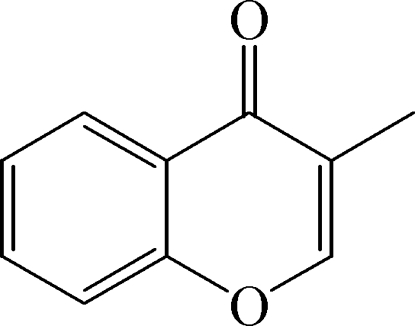


## Experimental

### 

#### Crystal data

C_10_H_8_O_2_
*M* *_r_* = 160.16
Triclinic, 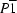

*a* = 6.5284 (13) Å
*b* = 7.2210 (14) Å
*c* = 8.9834 (18) Å
α = 75.137 (2)°
β = 78.169 (2)°
γ = 80.895 (2)°
*V* = 398.12 (14) Å^3^
*Z* = 2
Mo *K*α radiation
μ = 0.09 mm^−1^
*T* = 296 K
0.12 × 0.10 × 0.08 mm

#### Data collection

Bruker APEXII CCD diffractometer
Absorption correction: multi-scan (*SADABS*; Bruker, 2001 ▶) *T*_min_ = 0.989, *T*_max_ = 0.993
2771 measured reflections
1394 independent reflections
1143 reflections with *I* > 2σ(*I*)
*R*_int_ = 0.014

#### Refinement

*R*[*F* ^2^ > 2σ(*F* ^2^)] = 0.039
*wR*(*F* ^2^) = 0.116
*S* = 1.00
1394 reflections
111 parameters
H-atom parameters constrained
Δρ_max_ = 0.18 e Å^−3^
Δρ_min_ = −0.14 e Å^−3^

### 

Data collection: *APEX2* (Bruker, 2004 ▶); cell refinement: *SAINT-Plus* (Bruker, 2001 ▶); data reduction: *SAINT-Plus*; program(s) used to solve structure: *SHELXS97* (Sheldrick, 2008 ▶); program(s) used to refine structure: *SHELXL97* (Sheldrick, 2008 ▶); molecular graphics: *SHELXTL* (Sheldrick, 2008 ▶); software used to prepare material for publication: *SHELXTL*.

## Supplementary Material

Crystal structure: contains datablocks global, I. DOI: 10.1107/S1600536810020453/sj5009sup1.cif

Structure factors: contains datablocks I. DOI: 10.1107/S1600536810020453/sj5009Isup2.hkl

Additional supplementary materials:  crystallographic information; 3D view; checkCIF report

## Figures and Tables

**Table 1:** Hydrogen-bond geometry (Å, °)

*D*—H⋯*A*	*D*—H	H⋯*A*	*D*⋯*A*	*D*—H⋯*A*
C5—H5⋯O1^i^	0.93	2.70	3.4374 (19)	137
C7—H7⋯O2^ii^	0.93	2.69	3.3820 (19)	132

Symmetry codes: (i) 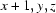; (ii) 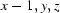.

**Fig. 1.**
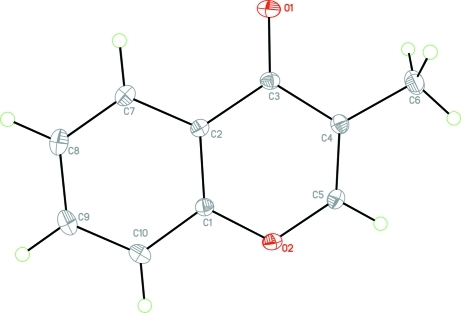
Structure of the title compound showing the atom numbering with displacement ellipsoids drawn at the 30% probability level. H atoms are shown as spheres of arbitrary radius.

**Fig. 2.**
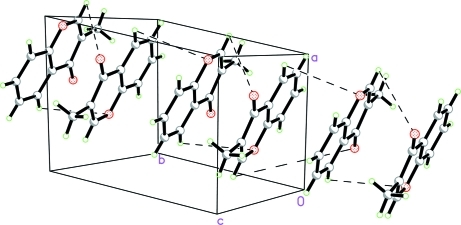
Crystal packing showing chains formed along the a axis via weak C—H···O contacts.

## supplementary crystallographic information

### Comment

The synthesis of chromanone derivatives has attracted continuous research interest due to their applications as vasodilator, anti-hypertensive, bronchodilator, heptaprotective, anti-tumor, anti-mutagenic, geroprotective and anti-diabetic agents (Shi *et al.*, 2004). Here, we describe the cystallization and structural characterization of the title compound.

As shown in Fig 1. the two fused six membered rings are coplanar with a mean deviation of 0.0261 (1) Å from the plane through the non-hydrogen atoms of the rings. The carbonyl and methyl substituents of the pyran ring also lie close to that plane with deviations of 0.0557 (1) and 0.1405 (1) Å, respectively. The C=O and C—O bond distances, 1.367 (2) and 1.231 (2)—1.355 (2) Å, respectively,  are in the normal range compared to reported chromanone derivatives (Takikawa & Suzuki, 2007; Patonay *et al.*, 2002; Alaniz & Rovis, 2005). In the crystal structure, chains along the *a* axis are formed *via* the weak C—H···O contacts.

### Experimental

3-methyl-4*H*-chromen-4-one powder (10 mmoL, 1.60 g) was purchased from Jinan Henghua Science & Technology Co. Ltd., dissolved in 20 ml ethanol and evaporated in an open flask at room temperature. One week later, colorless block like crystals of the title compound suitable for the X-ray analysis were obained. Anal. C_10_H_8_O_2_: C, 74.93; H, 5.00%. Found: C, 74.86; H, 4.89%.

### Refinement

Hydrogen atoms were placed in geometrically calculated positions (C—H 0.95 Å for aromatic and formyl, 0.99 Å for methylene and 0.98 Å for methyl) and included in the refinement in a riding motion approximation with *U*_iso_(H) = 1.2Ueq(C) [for methyl groups *U*_iso_(H) = 1.5Ueq(C)].

### Crystal data

**Table:**

C_10_H_8_O_2_	*Z* = 2
*M**_r_* = 160.16	*F*(000) = 168
Triclinic, *P*1	*D*_x_ = 1.336 Mg m^−^^3^
Hall symbol: -P 1	Mo *K*α radiation, λ = 0.71073 Å
*a* = 6.5284 (13) Å	Cell parameters from 1573 reflections
*b* = 7.2210 (14) Å	θ = 2.4–28.4°
*c* = 8.9834 (18) Å	µ = 0.09 mm^−^^1^
α = 75.137 (2)°	*T* = 296 K
β = 78.169 (2)°	Block, colorless
γ = 80.895 (2)°	0.12 × 0.10 × 0.08 mm
*V* = 398.12 (14) Å^3^	

### Data collection

**Table:**

Bruker APEXII CCD diffractometer	1394 independent reflections
Radiation source: fine-focus sealed tube	1143 reflections with *I* > 2σ(*I*)
graphite	*R*_int_ = 0.014
φ and ω scans	θ_max_ = 25.0°, θ_min_ = 2.4°
Absorption correction: multi-scan (*SADABS*; Bruker, 2001)	*h* = −7→7
*T*_min_ = 0.989, *T*_max_ = 0.993	*k* = −8→8
2771 measured reflections	*l* = −10→10

### Refinement

**Table:**

Refinement on *F*^2^	Secondary atom site location: difference Fourier map
Least-squares matrix: full	Hydrogen site location: inferred from neighbouring sites
*R*[*F*^2^ > 2σ(*F*^2^)] = 0.039	H-atom parameters constrained
*wR*(*F*^2^) = 0.116	*w* = 1/[σ^2^(*F*_o_^2^) + (0.065*P*)^2^ + 0.067*P*] where *P* = (*F*_o_^2^ + 2*F*_c_^2^)/3
*S* = 1.00	(Δ/σ)_max_ < 0.001
1394 reflections	Δρ_max_ = 0.18 e Å^−^^3^
111 parameters	Δρ_min_ = −0.13 e Å^−^^3^
0 restraints	Extinction correction: *SHELXL97* (Sheldrick, 2008), Fc^*^=kFc[1+0.001xFc^2^λ^3^/sin(2θ)]^-1/4^
Primary atom site location: structure-invariant direct methods	Extinction coefficient: 0.032 (10)

### Special details

**Table:**

Geometry. All e.s.d.'s (except the e.s.d. in the dihedral angle between two l.s. planes) are estimated using the full covariance matrix. The cell e.s.d.'s are taken into account individually in the estimation of e.s.d.'s in distances, angles and torsion angles; correlations between e.s.d.'s in cell parameters are only used when they are defined by crystal symmetry. An approximate (isotropic) treatment of cell e.s.d.'s is used for estimating e.s.d.'s involving l.s. planes.
Refinement. Refinement of *F*^2^ against ALL reflections. The weighted *R*-factor *wR* and goodness of fit *S* are based on *F*^2^, conventional *R*-factors *R* are based on *F*, with *F* set to zero for negative *F*^2^. The threshold expression of *F*^2^ > σ(*F*^2^) is used only for calculating *R*-factors(gt) *etc*. and is not relevant to the choice of reflections for refinement. *R*-factors based on *F*^2^ are statistically about twice as large as those based on *F*, and *R*- factors based on ALL data will be even larger.

### Fractional atomic coordinates and isotropic or equivalent isotropic displacement parameters (Å^2^)

**Table:**

	*x*	*y*	*z*	*U*_iso_*/*U*_eq_	
C1	0.5883 (2)	0.71757 (18)	1.05339 (16)	0.0417 (3)	
C2	0.4277 (2)	0.76708 (17)	0.96592 (15)	0.0390 (3)	
C3	0.4728 (2)	0.76636 (18)	0.79936 (15)	0.0427 (4)	
C4	0.6928 (2)	0.71855 (19)	0.73797 (16)	0.0455 (4)	
C5	0.8332 (2)	0.6710 (2)	0.83389 (17)	0.0503 (4)	
H5	0.9726	0.6398	0.7905	0.060*	
C6	0.7581 (3)	0.7246 (3)	0.56727 (18)	0.0700 (5)	
H6A	0.9074	0.6903	0.5445	0.105*	
H6B	0.7222	0.8524	0.5078	0.105*	
H6C	0.6864	0.6350	0.5400	0.105*	
C7	0.2263 (2)	0.8210 (2)	1.04176 (18)	0.0506 (4)	
H7	0.1150	0.8533	0.9864	0.061*	
C8	0.1897 (3)	0.8271 (2)	1.19601 (19)	0.0602 (4)	
H8	0.0549	0.8646	1.2443	0.072*	
C9	0.3541 (3)	0.7771 (2)	1.28035 (18)	0.0607 (5)	
H9	0.3291	0.7818	1.3851	0.073*	
C10	0.5524 (3)	0.7212 (2)	1.21027 (17)	0.0554 (4)	
H10	0.6621	0.6859	1.2671	0.066*	
O1	0.33452 (17)	0.80490 (17)	0.71782 (12)	0.0648 (4)	
O2	0.79069 (14)	0.66418 (15)	0.98916 (11)	0.0522 (3)	

### Atomic displacement parameters (Å^2^)

**Table:**

	*U*^11^	*U*^22^	*U*^33^	*U*^12^	*U*^13^	*U*^23^
C1	0.0424 (7)	0.0398 (7)	0.0441 (7)	−0.0053 (5)	−0.0089 (6)	−0.0103 (5)
C2	0.0379 (7)	0.0336 (6)	0.0444 (7)	−0.0056 (5)	−0.0078 (5)	−0.0057 (5)
C3	0.0444 (8)	0.0392 (7)	0.0440 (7)	−0.0064 (6)	−0.0133 (6)	−0.0031 (5)
C4	0.0487 (8)	0.0439 (7)	0.0421 (7)	−0.0055 (6)	−0.0062 (6)	−0.0080 (6)
C5	0.0397 (8)	0.0574 (9)	0.0522 (8)	−0.0016 (6)	−0.0028 (6)	−0.0162 (7)
C6	0.0782 (12)	0.0787 (12)	0.0445 (9)	−0.0009 (9)	−0.0016 (8)	−0.0108 (8)
C7	0.0418 (8)	0.0482 (8)	0.0594 (9)	−0.0046 (6)	−0.0063 (6)	−0.0106 (6)
C8	0.0552 (9)	0.0551 (9)	0.0635 (10)	−0.0085 (7)	0.0104 (7)	−0.0168 (7)
C9	0.0803 (12)	0.0573 (9)	0.0437 (8)	−0.0141 (8)	0.0018 (8)	−0.0164 (7)
C10	0.0673 (10)	0.0573 (9)	0.0461 (8)	−0.0084 (7)	−0.0180 (7)	−0.0129 (7)
O1	0.0559 (7)	0.0849 (8)	0.0544 (7)	−0.0039 (6)	−0.0253 (5)	−0.0076 (5)
O2	0.0403 (6)	0.0681 (7)	0.0517 (6)	0.0017 (5)	−0.0162 (4)	−0.0184 (5)

### Geometric parameters (Å, °)

**Table:**

C1—O2	1.3668 (17)	C6—H6A	0.9600
C1—C2	1.3854 (19)	C6—H6B	0.9600
C1—C10	1.387 (2)	C6—H6C	0.9600
C2—C7	1.3967 (19)	C7—C8	1.368 (2)
C2—C3	1.4657 (19)	C7—H7	0.9300
C3—O1	1.2312 (16)	C8—C9	1.388 (2)
C3—C4	1.450 (2)	C8—H8	0.9300
C4—C5	1.332 (2)	C9—C10	1.366 (2)
C4—C6	1.4961 (19)	C9—H9	0.9300
C5—O2	1.3548 (17)	C10—H10	0.9300
C5—H5	0.9300		
			
O2—C1—C2	121.70 (12)	H6A—C6—H6B	109.5
O2—C1—C10	116.53 (12)	C4—C6—H6C	109.5
C2—C1—C10	121.77 (14)	H6A—C6—H6C	109.5
C1—C2—C7	117.46 (13)	H6B—C6—H6C	109.5
C1—C2—C3	120.18 (13)	C8—C7—C2	121.23 (14)
C7—C2—C3	122.34 (13)	C8—C7—H7	119.4
O1—C3—C4	122.69 (13)	C2—C7—H7	119.4
O1—C3—C2	122.50 (13)	C7—C8—C9	119.91 (15)
C4—C3—C2	114.81 (11)	C7—C8—H8	120.0
C5—C4—C3	119.62 (13)	C9—C8—H8	120.0
C5—C4—C6	121.16 (14)	C10—C9—C8	120.34 (14)
C3—C4—C6	119.22 (13)	C10—C9—H9	119.8
C4—C5—O2	125.65 (13)	C8—C9—H9	119.8
C4—C5—H5	117.2	C9—C10—C1	119.28 (14)
O2—C5—H5	117.2	C9—C10—H10	120.4
C4—C6—H6A	109.5	C1—C10—H10	120.4
C4—C6—H6B	109.5	C5—O2—C1	117.89 (11)

### Hydrogen-bond geometry (Å, °)

**Table:**

*D*—H···*A*	*D*—H	H···*A*	*D*···*A*	*D*—H···*A*
C5—H5···O1^i^	0.93	2.70	3.4374 (19)	137
C7—H7···O2^ii^	0.93	2.69	3.3820 (19)	132

Symmetry codes: (i) *x*+1, *y*, *z*; (ii) *x*−1, *y*, *z*.
